# Mutual interaction between endothelial cells and mural cells enhances BMP9 signaling in endothelial cells

**DOI:** 10.1242/bio.020503

**Published:** 2017-03-15

**Authors:** Yuki Tachida, Nanae Izumi, Toyo Sakurai, Hideki Kobayashi

**Affiliations:** 1 Pain and Neuroscience Laboratories, R&D Division, Daiichi Sankyo Co., Ltd., Tokyo 140-8710, Japan; 2 End-Organ Disease Laboratories, R&D Division, Daiichi Sankyo Co., Ltd., Tokyo 140-8710, Japan; 3 Hit Discovery and Cell Processing Research Group Biological Research Department, Daiichi Sankyo RD Novare Co., Ltd., Tokyo 134-8630, Japan

**Keywords:** BMP9, Mural cells, Cell–cell interaction, HHT, Vascular integrity

## Abstract

Hereditary hemorrhagic telangiectasia is characterized by the formation of abnormal vascular networks and caused by the mutation of genes involved in BMP9 signaling. It is also known that the interaction between endothelial cells (ECs) and mural cells (MCs) is critical to maintain vessel integrity. However, it has not yet fully been uncovered whether the EC–MC interaction affects BMP9 signaling or not. To elucidate this point, we analyzed BMP9 signaling in a co-culture of several types of human primary culture ECs and MCs. The co-culture activated the Notch pathway in both types of cells in a co-culture- and BMP9-dependent manner. In HUVECs, the genes induced by BMP9 were significantly and synergistically induced in the presence of pericytes, fibroblasts or mesenchymal stem cells. The synergistic induction was greatly reduced in a non-contact condition. In fibroblasts, PDGFRB expression was potently induced in the presence of HUVECs, and BMP9 additively increased this response. Taken together, these results suggest that the EC–MC interaction potentiates BMP9 signaling both in ECs and MCs and plays a critical role in the maintenance of proper vessel functions.

## Abstract (precis)

**Summary:** A mutual interaction between endothelial cells and mural cells enhances BMP9 signaling in endothelial cells, with implications for the maintenance of vascular integrity and vascular disease research.

## INTRODUCTION

Recently, an increasing amount of evidence has shown that the mutual interaction between endothelial cells (ECs) and mural cells (MCs), microvascular periendothelial mesenchymal cells that cover ECs in vessels, is pivotal to the maintenance of vessel integrity (Armulik et al., 2011; Gaengel et al., 2009; Geevarghese and Herman, 2014; van Dijk et al., 2015). Aberrant interaction between the two types of cells is associated with several human diseases, such as diabetic retinopathy, venous malformation and hereditary stroke (Dziewulska and Lewandowska, 2012; Gaengel et al., 2009; Trost et al., 2016). The interaction between ECs and MCs regulates various cellular functions and responses of each cell, such as gene expression, cell proliferation, and cell differentiation, and is regulated in a direct, paracrine, and autocrine fashion (Armulik et al., 2011; Dziewulska and Lewandowska, 2012). For example, Notch signaling plays an important role in the differentiation, maturation, and function of vascular smooth muscle cells (vSMCs) (Fouillade et al., 2012). The mutation of NOTCH3 causes cerebral autosomal dominant arteriopathy with subcortical infarcts and leukoencephalopathy (CADASIL), a disease related to stroke and dementia, due to the degeneration of vSMCs (Joutel et al., 1996). NOTCH3 knockout mice have been shown to reveal an abnormal maturation of vSMCs and failure to properly interact with ECs (Domenga et al., 2004). Notch pathway activation in vSMCs through NOTCH3 is stimulated by Jagged-1 expressed in ECs, which induces expression of platelet derived growth factor receptor β (PDGFRβ) and maintains the proper response to PDGF, an important regulator of vSMC proliferation and differentiation (Jin et al., 2008). Signals from ECs to MCs are essential to maintain adequate vessel function.

Hereditary hemorrhagic telangiectasia (HHT) is characterized by the formation of abnormal vascular networks inducing arteriovenous malformation, which results in hemorrhage and stroke (Govani and Shovlin, 2009). HHT is caused by a heterogeneous loss-of-function mutation in genes involved in bone morphogenic protein-9 (BMP9) and bone morphogenic protein-10 (BMP10) signaling. Two major genes responsible for HHT are activin A receptor like type 1 (ALK1/ACVRL1) (Johnson et al., 1996), which is a type I receptor of BMP9/10, and endoglin (ENG) (McAllister et al., 1994), which is a co-receptor of ALK1. BMP9 binds to ALK1 expressed in ECs, signals through smad1/5/8 activation, and regulates vascular quiescence and angiogenesis (David et al., 2007, 2008; Scharpfenecker et al., 2007) through regulating various kinds of gene expression, such as matrix gla protein (MGP) (Bostrom et al., 2004; Yao et al., 2012), BMP binding endothelial regulator (BMPER) (Yao et al., 2012), and transmembrane protein 100 (TMEM100) (Somekawa et al., 2012). These BMP9-responsive genes are known to regulate endothelial differentiation, promote angiogenesis, inhibit vascular calcification, and protect endothelial cells from cell death (Moon et al., 2015; Moreno-Miralles et al., 2011; Yao et al., 2011). Growing evidence supports the notion that impaired BMP9/10 signaling caused by heterogeneous mutation of ALK1 or ENG results in the decrease of BMP9/10-responsive gene expression and this causes HHT phenotypes. Normalization of BMP9/10 signaling in HHT patient vessels would be a promising way to treat HHT. Therefore, a deeper understanding of the regulating mechanisms of BMP9 signaling is pivotal to accomplishing this aim.

In this report, we analyzed the effects of the EC–MC interaction on BMP9 signaling in an *in vitro* co-culture assay of HUVECs and mural cells. The results suggest that the direct cell–cell interaction of ECs and MCs potentiates BMP9 signaling. This synergistic effect between the EC–MC interaction and BMP9 signaling could provide new insights into development of therapeutic agents for HHT.

## RESULTS

### Notch pathway is synergistically activated by BMP9 and the EC–MC interaction

**Fig. 1.**
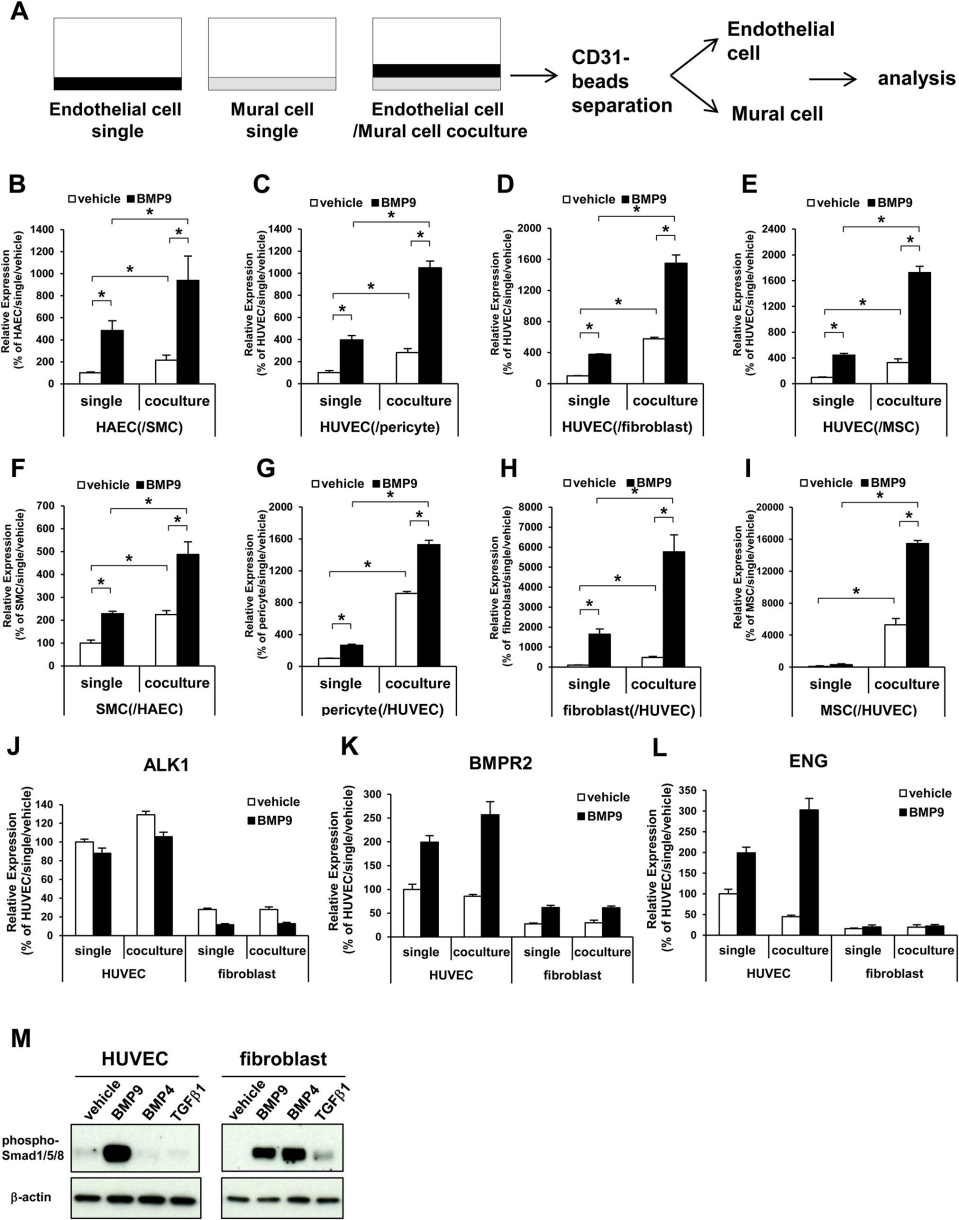
**EC–MC interaction potentiates HEY1 expression by BMP9.** (A) Diagrammatic representation of the co-culture experiment. The co-cultured endothelial cells (ECs, black) and mural cells (MCs, gray) were separated by magnetic beads and used for qRT-PCR experiments. (B-I) The cells were co-cultured in four combinations, HAEC/SMC (B,F), HUVEC/pericyte (C,G), HUVEC/fibroblast (D,H) and HUVEC/MSC (E,I). The expression level of HEY1 in ECs (B-E) and MCs (F-I) were analyzed (*n*=3 biological replicates). (J-L) The expression analysis of BMP9 receptors ALK1 (J), BMPR2 (K) and ENG (L) in HUVECs and fibroblasts (*n*=3 biological replicates). All values are mean±s.d. *P<0.05; Student's *t*-test. (M) Western blot analysis of phospho-smad1/5/8. The single-cultured cells were treated with BMP9 (10 ng/ml), BMP4 (100 ng/ml) or TGF-β (10 ng/ml) for 1 h. Representative image from three independent experiments were shown.

To evaluate the effects of the interaction between endothelial cells (ECs) and mural cells (MCs) on the bone morphogenic protein-9 (BMP9) pathway, we analyzed the BMP9 signaling in the co-culture of several types of human primary culture ECs and MCs. Human aortic endothelial cells (HAECs) were co-cultured on human smooth muscle cells (SMCs) and human umbilical vein endothelial cells (HUVECs) were co-cultured on human primary culture pericytes (pericytes), human primary culture fibroblasts (fibroblasts), or mesenchymal stem cells (MSCs), and treated with BMP9. After BMP9 stimulation, the ECs and MCs were separated by using CD31-magnetic beads and gene expression in each of the cells was evaluated by quantitative PCR (qPCR) (Fig. 1A). Since it has been demonstrated that the Notch pathway is an important regulator of the EC–MC interaction and also affects BMP9 signaling and functions (Domenga et al., 2004; Gaengel et al., 2009; Ricard et al., 2012; Larrivee et al., 2012), the activation status of the Notch pathway was analyzed. The expression of hairy/enhancer-of-split related with YRPW motif protein 1 (HEY1), which is induced downstream of Notch activation, was induced by BMP9 in ECs (Fig. 1B-E) and MCs (Fig. 1F-I). The co-culture itself also amplified Notch signaling activation. Importantly, BMP9 stimulation in the co-culture condition dramatically and synergistically potentiated HEY1 induction (Fig. 1B-I). Because this trend of HEY1 expression change was observed in all four combinations of ECs and MCs, we decided to use the co-culture of HUVECs with fibroblasts to conduct further analysis because of its easiness to handle. Then, the expressions of genes involved in the BMP9 signaling pathway were also analyzed in the HUVEC and fibroblasts co-culture. Activin A receptor like type 1 (ALK1), a type I receptor of BMP9 (Fig. 1J), BMP type-II receptor (BMPR2), a type II receptor of BMP9 (Fig. 1K), and endoglin (ENG), a co-receptor of ALK1 (Fig. 1L), were all expressed in both cell types. The expression levels of these genes were higher in the HUVECs than in the fibroblasts. In a single culture condition, BMP9 induced phosphorylation of smad1/5/8 in both HUVECs and fibroblasts (Fig. 1M). These data indicated that BMP9 is able to directly activate the Notch pathway in each type of cell. By contrast, the interaction between HUVECs and fibroblasts drastically and synergistically potentiated activation of the Notch pathway by BMP9.

**Fig. 2.**
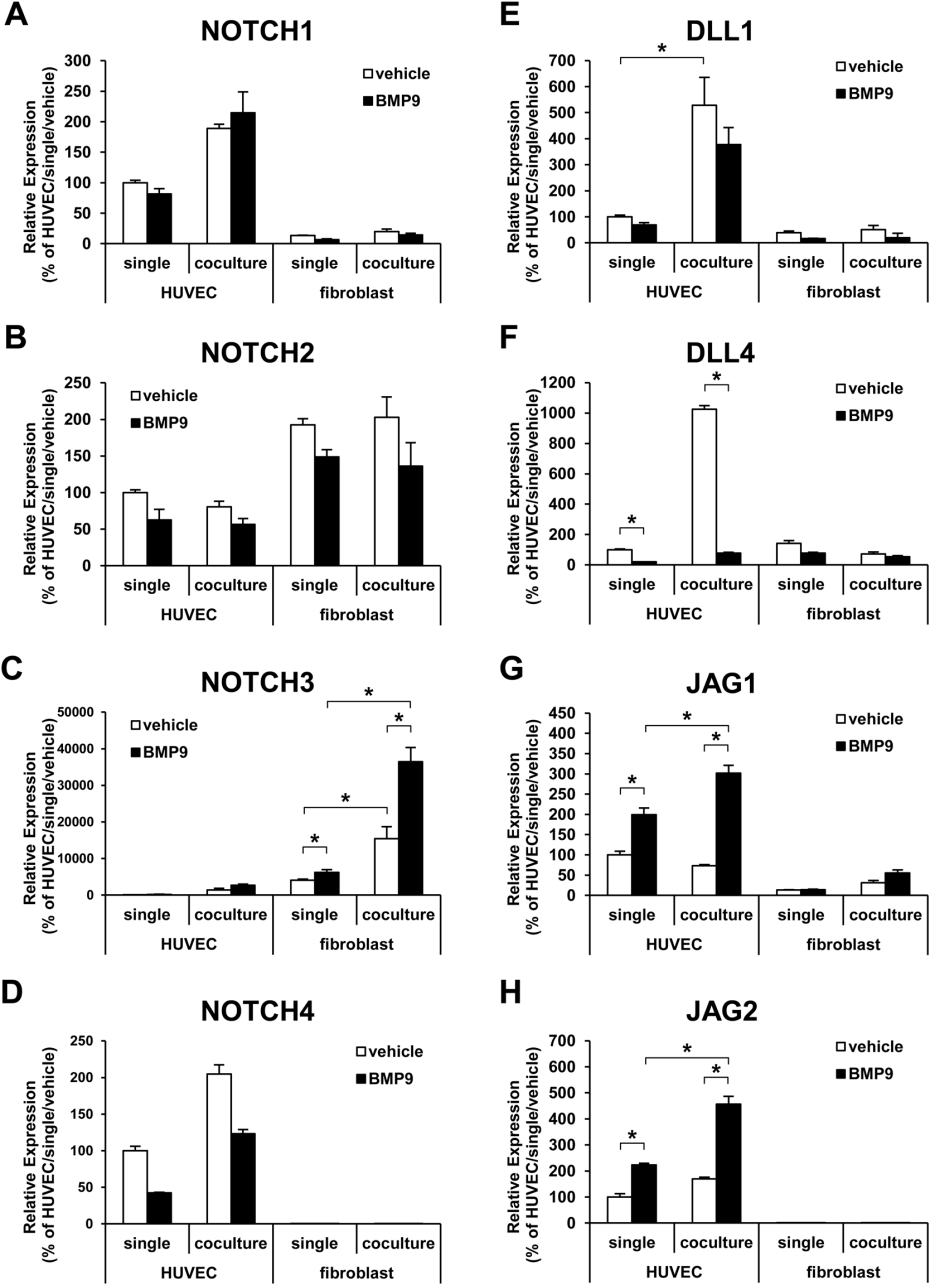
**Expression change of Notch receptors and Notch ligands by co-culture or BMP9 treatment in HUVECs and fibroblasts.** The expression of Notch (NOTCH1-4) (A-D) and Notch ligands (DLL1, DLL4, JAG1, JAG2) (E-H) were analyzed by qRT-PCR. The cells were cultured in a single- or co-culture condition and treated with (black bar) or without (white bar) BMP9 (*n*=3 biological replicates). The expression of DLL3 was below detectable levels and not shown. All values are mean±s.d. *P<0.05; Student's *t*-test.

Next, gene expression of the Notch receptors (Notch 1-4) and Notch ligands (Delta like-1, 3, 4; Jagged-1, 2) in each cell type was analyzed in single and co-cultured conditions (Fig. 2A-H). As reported in a previous study (Liu et al., 2009), NOTCH3 was expressed mainly in fibroblasts and its expression was induced by ECs (Fig. 2C). Furthermore, NOTCH3 was potently induced by BMP9 in the condition of EC co-culture (Fig. 2C). The expression of Notch ligands was relatively higher in HUVECs than in fibroblasts (Fig. 2E-H). In HUVECs, the expression of DLL1 was induced by the fibroblast-co-culture (Fig. 2E) and the expression of JAG1 and JAG2 was induced by BMP9 (Fig. 2G,H). The expression of DLL4, which is known to be induced during the angiogenic state, was down regulated by BMP9 (Fig. 2F). These data indicate that the dramatic activation of the Notch pathway in fibroblasts by BMP9 in the co-culture condition was caused by the synergistic consequence of induction of Notch ligands in HUVECs, such as JAG1 and JAG2, induced by BMP9, and induction of NOTCH3 in fibroblasts induced by both BMP9 and the HUVEC co-culture.

### BMP9 response in ECs was synergistically potentiated by direct interaction with MCs

**Fig. 3.**
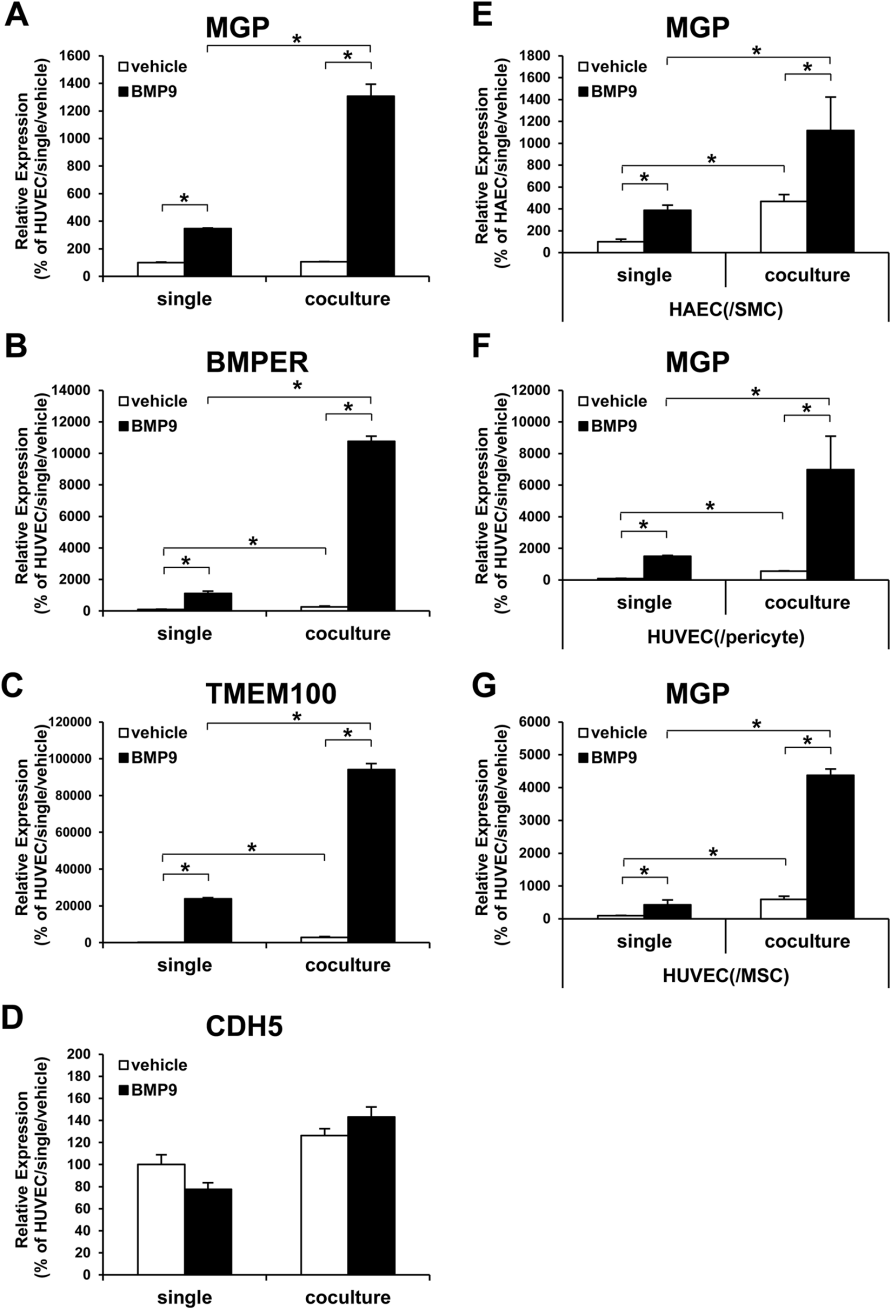
**MCs synergistically enhanced expression of BMP9-responsive genes induced by BMP9 in HUVECs.** The expression of BMP9-responsive genes was analyzed by qRT-PCR (*n*=3 biological replicates). HUVECs were cultured in single-culture condition or in co-culture condition with fibroblasts (A-D), pericytes (F) or MSC (G), and HAECs were cultured in single-culture condition or in co-culture condition with SMC (E). These cells were treated with (black bar) or without (white bar) BMP9. All values are mean±s.d. *P<0.05; Student's *t*-test.

To further investigate the synergistic interplay between the EC–MC interaction and BMP9 signaling, first we focused on the response in ECs. In the HUVEC and fibroblast co-culture, the expression of genes known to be induced by BMP9 (BMP9-responsive genes) was analyzed. Matrix gla protein (MGP), which is an important regulator of vascular calcification and has been reported as a BMP9-responsive gene (Yao et al., 2012), was induced by BMP9 at about threefold the single-culture condition, whereas in the co-culture condition, the induction by BMP9 reached 12-fold (Fig. 3A). The same synergistic inductions were also observed in BMP-binding endothelial regulator (BMPER) and transmembrane protein 100 (TMEM100). BMPER has also been reported to be induced by BMP9 (Yao et al., 2012). We confirmed induction of BMPER by BMP9 in the single-culture condition and found a dramatic induction by BMP9 in the co-culture condition (Fig. 3B). TMEM100 has been reported to be one of the most sensitive BMP9-responsive genes (Somekawa et al., 2012), and we indeed found that TMEM100 expression was induced over 200-fold by BMP9 in the single culture. Again, the co-culture on fibroblasts dramatically potentiated TMEM100 induction by BMP9 up to 900-fold (Fig. 3C). The expression level of VE-cadherin, an endothelial cell marker, was not affected by either co-culture or BMP9 (Fig. 3D), confirming that the synergistic effects are not caused by the change of differentiation status of HUVECs. To confirm that the synergistic activation effect on BMP-9-responsive genes is not limited only to the combination of HUVEC and fibroblasts, the co-culture of HAECs with SMC, HUVECs with pericytes, and HUVECs with hMSCs, was also conducted. The same synergistic induction of MGP was observed in the combination of HAECs and SMCs (Fig. 3E), HUVECs and pericytes (Fig. 3F), and HUVECs and MSCs (Fig. 3G). These data indicate that the EC–MC interaction drastically potentiates the BMP9 response in ECs.

**Fig. 4.**
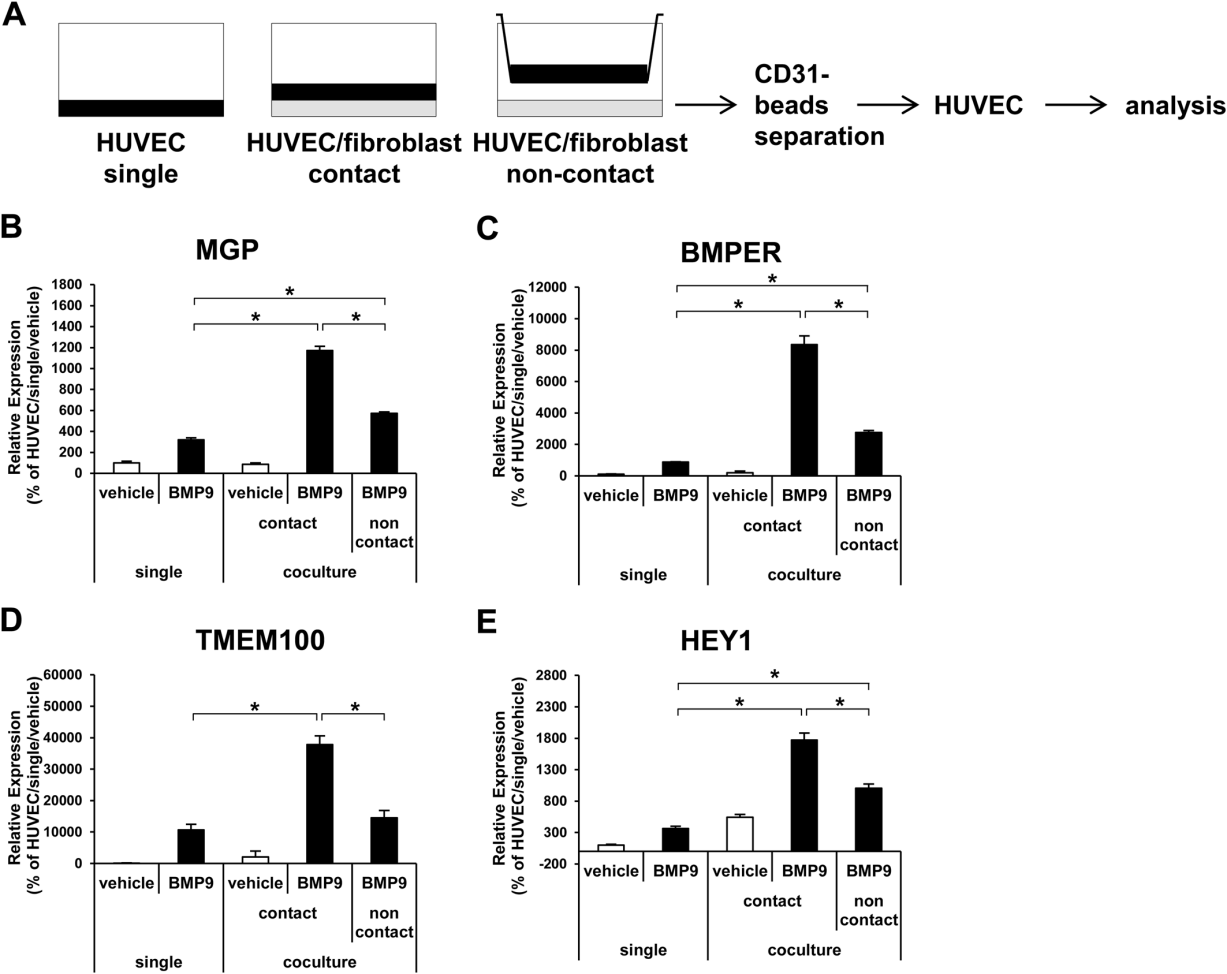
**Direct cell–cell interaction is required for the synergistic effects in HUVECs.** (A) Diagrammatic representation of the non-contact co-culture experiment. (B-E) Expression analysis of BMP9-responsive genes and HEY1 in HUVECs by qRT-PCR. The HUVECs were cultured in a single- or co-culture with fibroblasts in a contact or non-contact condition, and then treated with (black bar) or without (white bar) BMP9 (*n*=3 biological replicates). All values are mean±s.d. *P<0.05; Student's *t*-test.

**Fig. 5.**
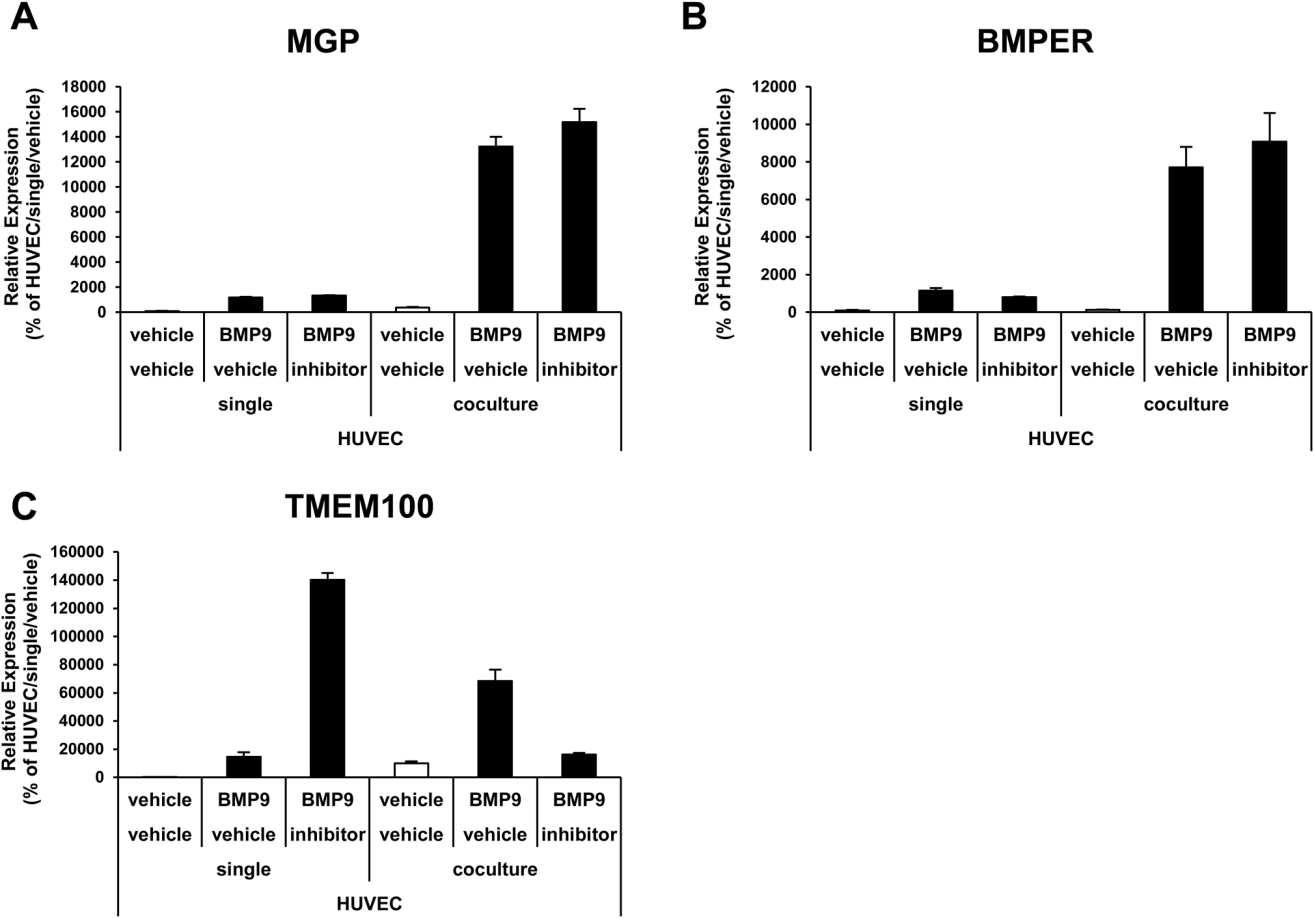
**Notch inhibition did not affect the synergistic effects in HUVECs.** (A-C) Expression analysis of BMP9-responsive genes in HUVECs by qRT-PCR. The HUVECs were cultured in a single- or co-culture conditions and treated with (black bar) or without (white bar) BMP9 in the absence or presence of Notch inhibitor (*n*=3 biological replicates). All values are mean±s.d.

In order to elucidate the mechanisms of the synergistic effects, the importance of direct interaction between ECs and MCs was evaluated. A non-contact co-culture was conducted by culturing HUVECs in a Boyden chamber in the presence of fibroblasts in the lower chamber (Fig. 4A). The synergistic effects on the expression of MGP, BMPER, TMEM100 and HEY1 were greatly reduced in the non-contact culture (Fig. 4B-E). Finally, to analyze the involvement of the Notch pathway in the synergistic effects, the effects of a Notch inhibitor were analyzed. The Notch inhibitor did not affect the synergistic effects on MGP and BMPER (Fig. 5A,B). Although the expression of TMEM100 was clearly suppressed to the basal level by Notch inhibition in the co-culture condition, in the single-culture condition the expression of TMEM100 was strangely induced by Notch inhibition (Fig. 5C). This indicated that the Notch inhibition itself greatly changes TMEM100 expression, and this made it difficult to conclude the importance of the Notch pathway to the synergistic effects in TMEM100. Taken together, these data suggest that the EC–MC interaction synergistically potentiates BMP9 response in ECs through direct contact between the two types of cells.

### Notch pathway activation in MCs was induced by Notch ligand induction in ECs by BMP9

**Fig. 6.**
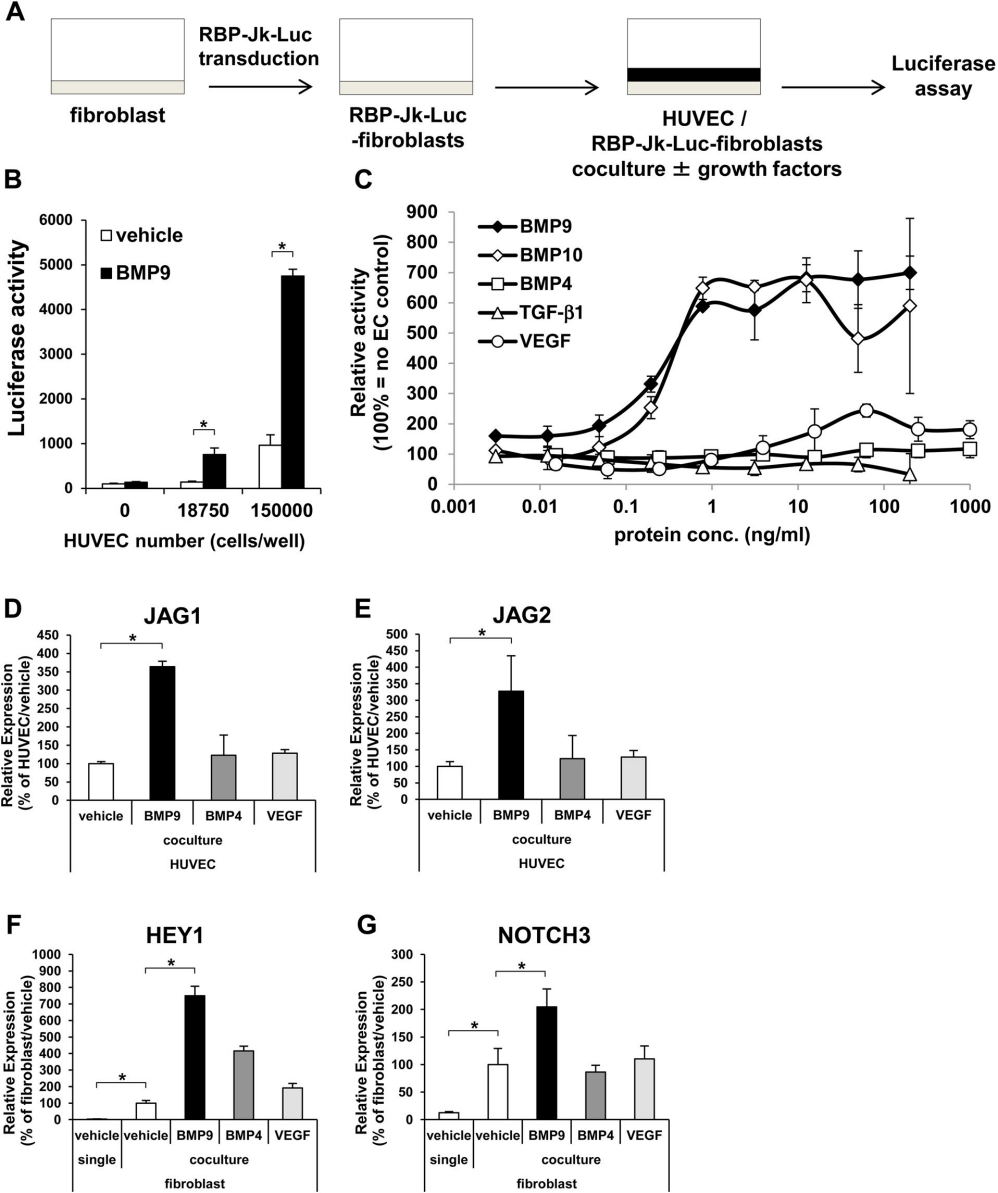
**Notch pathway activation in MCs was in an EC- and BMP9-dependent fashion.** (A) Diagrammatic representation of RBP-Jk–Luc assay. The Notch reporter-transduced fibroblasts (RBP-Jk–Luc fibroblasts) (gray) were co-cultured with HUVECs (black). (B) The RBP-Jk–fibroblasts were cultured with or without HUVECs and treated with (black bar) and without (white bar) BMP9 (*n*=3 biological replicates). (C) RBP-Jk–Luc–fibroblasts/HUVEC co-culture were treated with various growth factors at a range of concentrations (*n*=3 biological replicates). (D-G) Gene expression analyses of JAG1 and JAG2 in HUVECs (D,E) and HEY1 and NOTCH3 in fibroblasts (F,G) were conducted by qRT-PCR. The cells were treated with indicated growth factors (*n*=3 biological replicates). All values are mean±s.d. *P<0.05; Student's *t*-test.

Next, we analyzed the response in MCs. Since, as mentioned above, the importance of the Notch pathway in the regulation of mural cell functions is well-known (Domenga et al., 2004; Jin et al., 2008; Liu et al., 2009) and we found a drastic induction of HEY1 expression in fibroblasts (Fig. 1C), we analyzed the Notch signals in fibroblasts. To monitor the activity of the Notch signal transduction pathways solely in fibroblasts, a RBP-Jk luciferase reporter construct was transduced in fibroblasts. RBP-Jk protein is a transcription factor, and a direct downstream modulator of Notch signaling. Fibroblasts transduced with RBP-Jk–Luc were co-cultured with HUVECs and stimulated with BMP9 (Fig. 6A). BMP9 activation of the Notch signaling pathway in fibroblasts was in a HUVEC/EC-dependent manner (Fig. 6B). The EC50 value of BMP9 was ∼0.3 ng/ml (Fig. 6C). Notch signaling was also activated by bone morphogenic protein-10 (BMP10), which is known to activate ALK1/ENG signaling (Levet et al., 2015; Ricard et al., 2012), but was not activated by other vasoactive factors, such as bone morphogenic protein 4 (BMP4), transforming growth factor-β (TGF-β), and vascular endothelial growth factor (VEGF) (Fig. 6C). In the co-culture condition, the expression of JAG1 (Fig. 6D) and JAG2 (Fig. 6E) in HUVECs were induced by BMP9, but not by BMP4 and VEGF. And in fibroblasts, HEY1 (Fig. 6F) and NOTCH3 (Fig. 6G) were strongly induced by HUVECs, and BMP9 further enhanced this induction. Interestingly, BMP4 induced HEY1 expression but did not induce NOTCH3 (Fig. 6F,G). These data suggest that Notch ligands induced by BMP9 in HUVECs stimulate the Notch signal in fibroblasts.

**Fig. 7.**
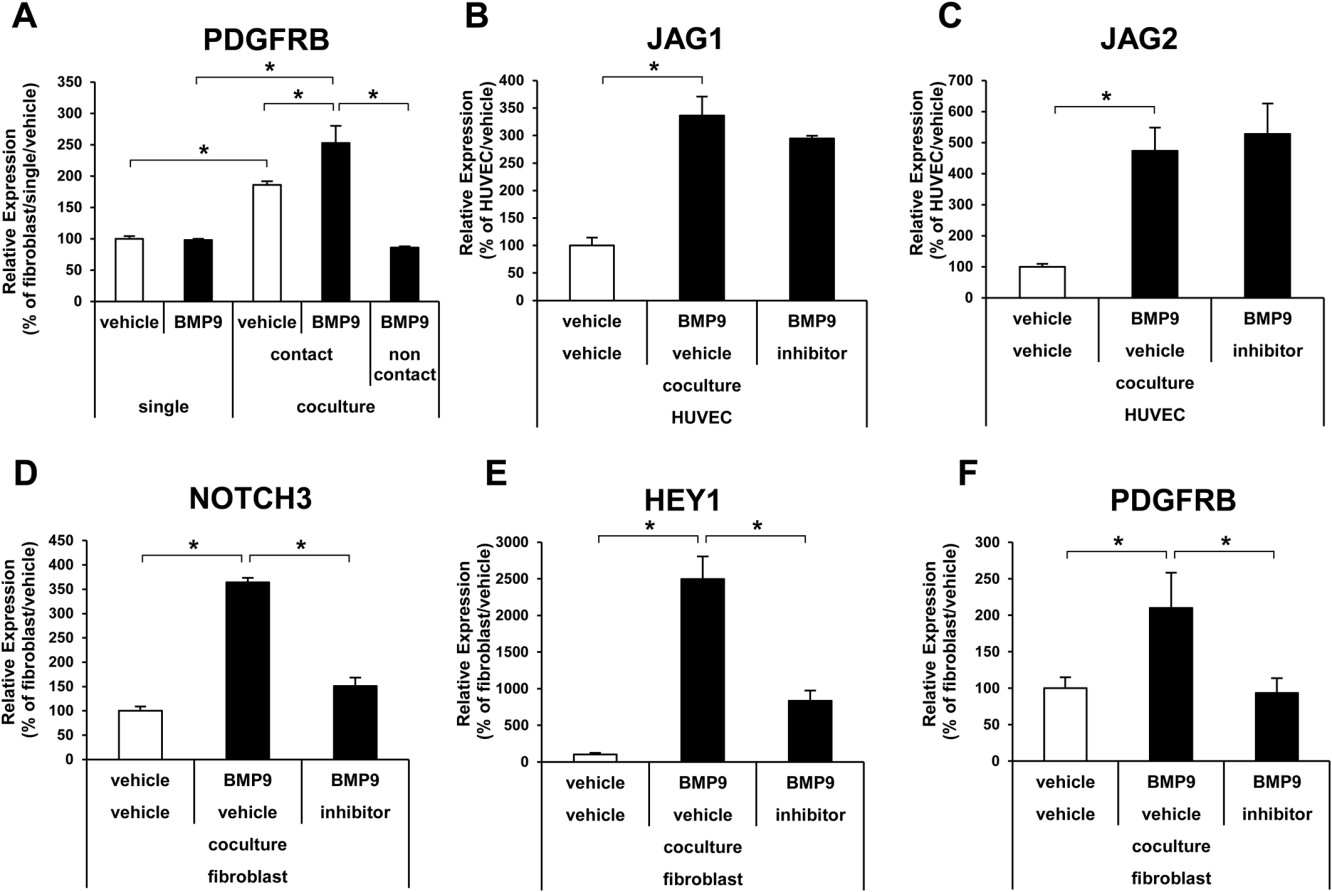
**Notch ligand induction in ECs enhanced the effect of BMP9 on MCs.** (A) Expression analysis of PDGFRB in fibroblasts by qRT-PCR. The fibroblasts were cultured in a single culture or co-culture with HUVECs in a contact or non-contact condition, and then treated with (black bar) or without (white bar) BMP9 (*n*=3 biological replicates). (B-F) Effects of Notch inhibition on gene expression. HUVECs and fibroblasts were co-cultured and treated with (black bar) and without (white bar) BMP9 in the presence or absence of Notch inhibitor IX (inhibitor) (*n*=3 biological replicates). All values are mean±s.d. *P<0.05; Student's *t*-test.

To further investigate the biological outcome of the EC–MC interaction on BMP9 signaling in fibroblasts, the expression of platelet-derived growth factor receptor beta (PDGFRB) was analyzed. PDGFRB has been reported to be upregulated by Notch activation and augments response to platelet derived growth factor (PDGF) in vSMCs (Jin et al., 2008). The HUVEC–fibroblast co-culture drastically upregulated PDGFRB expression in fibroblasts, and BMP9 slightly but significantly increased the response (Fig. 7A). In the non-contact co-culture in the Boyden chamber, the expression of PDGFRB was decreased to the same level as that of the single culture (Fig. 7A). In the direct co-culture condition, the treatment of Notch inhibitor did not affect the induction of JAG1 and JAG2 by BMP9 in HUVECs (Fig. 7B,C), but suppressed the induction of NOTCH3, HEY1, and PDGFRB in fibroblasts (Fig. 7D-F). Taken together, these data indicate that BMP9 induced PDGFRB expression in MCs through the induction of Notch ligands in ECs.

## DISCUSSION

**Fig. 8.**
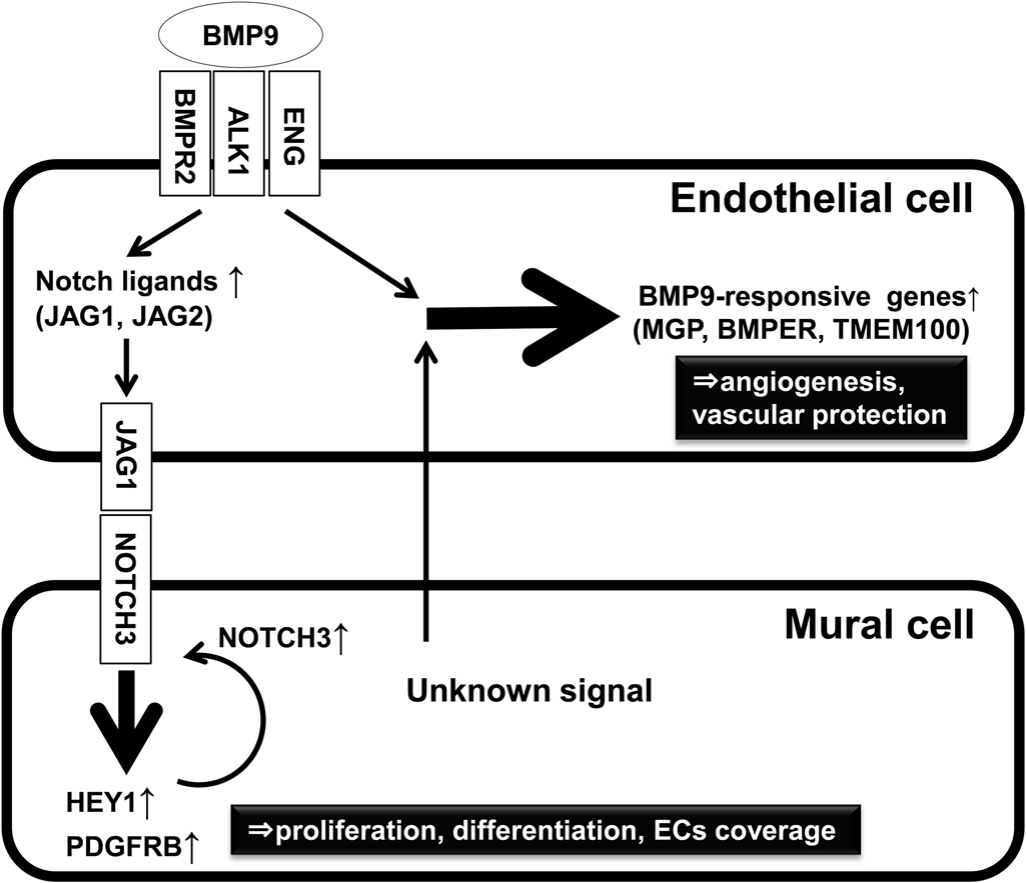
**Working model for BMP9 signaling potentiation by EC–MC interaction.** The data suggest that in MCs NOTCH3 induced by the EC–MC interaction is activated by Notch ligands in ECs induced by BMP9. The additive effects result in higher PDGFRB induction, which leads to MC proliferation, differentiation, and proper ECs coverage. In ECs (right part), BMP9 signaling is strongly potentiated by unknown signaling conveyed by the direct EC–MC interaction. The synergistic effects result in dramatic induction of BMP9-responsive genes, which function to promote angiogenesis and maintain proper vessel integrity.

The data presented here suggest that the mutual interaction between ECs and MCs potentiates BMP9 signaling in both cell types. Fig. 8 shows our working hypotheses. In MCs, NOTCH3 induced by the EC–MC interaction was activated by Notch ligands induced by BMP9 in ECs. The additive increase of Notch ligand and Notch receptor resulted in a higher PDGFRB induction, which would lead to MC proliferation, differentiation, and proper coverage of ECs. In ECs, BMP9 signaling was strongly potentiated by unknown signaling conveyed by the direct EC–MC interaction. The synergistic effects resulted in a dramatic induction of BMP9-responsive genes, which would function to promote angiogenesis and protect ECs from cell death. Taken together, BMP9 signaling potentiation by the EC–MC interaction would be critical to the maintenance of proper vascular integrity.

Proper interaction between endothelial cells (ECs) and mural cells (MCs) is vital to the maintenance of normal vessel properties and functions (Armulik et al., 2011; Gaengel et al., 2009; Geevarghese and Herman, 2014; van Dijk et al., 2015). A number of genetic mutations are reported to cause impaired EC–MC interaction and are linked to human diseases, such as ALK1 or ENG in hereditary hemorrhagic telangiectasia (HHT) (Johnson et al., 1996; McAllister et al., 1994), BMPR2 and ALK1 in pulmonary artery hypertension (PAH) (Lane et al., 2000; Trembath et al., 2001), NOTCH3 in cerebral autosomal dominant arteriopathy with subcortical infarcts, and leukoencephalopathy (CADASIL) (Joutel et al., 1996). Interestingly, some of these genes are involved in BMP9 signaling; ALK1, BMPR2 and ENG form the receptor complex of BMP9 (Peacock et al., 2016; Tillet and Bailly, 2014). It has been reported that NOTCH3 expressed in MCs is activated by JAG1, which is induced by BMP9 in ECs (Liu et al., 2009; Ricard et al., 2012). So, it is reasonable to speculate that BMP9 signaling and EC–MC have a close interaction with one another. In this report, we found that the BMP9 response in ECs is dramatically potentiated through the interaction of the ECs and MCs (Figs. 1 and 3), and also that the signal from ECs to MCs is significantly enhanced through BMP9 signaling (Fig. 7). A limitation of our research is that we mainly used fibroblasts as MCs and we showed only *in vitro* results. In order to further strengthen this hypothesis, it is necessary to conduct *in vivo* studies and *in vitro* experiments in the condition that more accurately reproduce *in vivo* vasculature using other types of cells.

The synergistic action of BMP9 and the EC–MC interaction are especially drastic in ECs. The expression level of MGP, BMPER and TMEM100 induced by BMP9 in the co-culture condition was five- to 10-fold higher than that in the single-culture condition (Fig. 3A-C,E-G). MGP is known as a potent inhibitor of vascular calcification, and mutation of MGP has been linked to Keutel syndrome, which is characterized by abnormal calcium deposition in peripheral stenosis of the pulmonary artery (Munroe et al., 1999). BMPER is a binder of BMPs and has been reported to bind to and inhibit BMP9 (Yao et al., 2012). In the vascular system, BMPER regulates angiogenesis through modulating BMP signaling and BMPER deficient mice show abnormal angiogenesis (Moreno-Miralles et al., 2011). TMEM100 is identified as one of the most sensitive BMP9-responsive genes in ECs (Somekawa et al., 2012) and it has been found that the decreased TMEM100 expression is linked to the development of the vascular pathology of HHT (Moon et al., 2015). Since all of these genes function as vascular-protective factors, it is possible that the interaction between ECs and MCs potentiates the induction of these genes by BMP9 in human vessels and maintains proper vascular functions. It has also been reported that several diseases, including HHT and PAH, are caused by impaired BMP9 signaling. Enhancement of BMP9 signaling in a PAH model mouse, which is caused by the heterozygous mutation of BMPR-II, reversed the PAH phenotype (Long et al., 2015). Therefore, enhancement of BMP9 signaling in vasculature is a promising therapeutic strategy for PAH and HHT. The potentiation of BMP9 signaling by normalizing the EC–MC interaction could be one of the promising and effective ways to treat vascular pathologies such as PAH and HHT. Further investigation is required to accomplish this goal and it is critically important to know whether the expression of these genes is decreased in a disease condition.

The precise mechanism of the synergistic action we have reported here is still unclear. Besides the vascular functions, BMP9 is also known as one of the most osteogenic BMPs (Luu et al., 2007). Although the specific mechanism of osteogenic action of BMP9 has not been fully uncovered, several papers report the existence of synergic factors such as IGF2 (Chen et al., 2010), Wnt3a (Zhang et al., 2013), all-trans retinoic acid (Liu et al., 2014), and growth hormone (Huang et al., 2012). Impressively, all of these factors work in a paracrine fashion. In ECs we found that direct interaction between ECs and MCs, but not trophic factor, is critical for the synergistic action (Fig. 4). The most probable mechanism accounting for the direct interaction would be regulation by Notch signaling. Actually, it has been reported that Notch signaling is an important regulator of BMP9 signaling (Morikawa et al., 2011) and BMP9/ALK1 and DLL4 synergize to activate HEY1 and HEY2 in a mutual interaction among ECs (Larrivee et al., 2012). However, we did not confirm the involvement of the Notch pathway in the synergistic effects in ECs (Fig. 5). The elucidation of the underlying mechanism of the synergistic action would provide important clues to understanding the BMP9 signaling. It would be an interesting approach to analyze the osteogenic action of BMP9 by searching for synergistic factors while focusing on the direct cell–cell contact. It could also provide an effective way to cure BMP9-signaling-related human diseases.

The results shown here suggest that the mutual interaction between ECs and MCs potentiates BMP9 signaling in both cells. Since potentiation of BMP9 signaling might be an effective way to cure PAH and HHT, it would also be a promising therapeutic approach that enhances the EC–MC interaction or conveys corresponding signaling to treat those diseases. Although confirmation in *in vivo* vasculature and further precise analysis in an appropriate EC–MC combination is needed to prove this hypothesis, the data shown here provide the first clue to elucidate this point.

## MATERIALS AND METHODS

### Cell culture

Human mesenchymal stem cells (MSCs) (Lonza) were cultured with Dulbecco's Modified Eagle Medium (DMEM) (Life Technologies) containing 10% FBS (Life Technologies) and Antibiotic-Antimitotic (Life Technologies). Human umbilical vein endothelial cells (HUVECs) (Kurabo) and Human dermal fibroblasts (Kurabo) were cultured with HuMedia-EG2 (Kurabo). Human pericytes (PromoCell) were cultured with pericytes growth medium (PromoCell). Human aortic smooth muscle cells (SMC) (Gibco) were cultured with Medium 231 containing smooth muscle growth supplement (Gibco).

### Co-culture and Transwell

Feeder cells (fibroblasts, MSCs, SMCs and pericytes) were seeded into a 6-well plate and cultured until confluent. Then, 1.5×10^5^ endothelial cells (HUVECs or HAECs) were seeded into these feeder cells or into a collagen-coated Boyden chamber (0.4 µm pore, BD). The next day, the culture media were replaced with HuMedia and the cells were treated with recombinant human BMP9 (10 ng/ml) (R&D systems). After an 18-22 h incubation with BMP9, the cells were harvested by trypsin/EDTA treatment (Life Technologies) and HUVECs were separated from fibroblasts or MSCs by magnetic cell sorting (MACS) using a CD31 MicroBead Kit (Miltenyi Biotech) following the manufacturer's instructions. For Notch pathway inhibition, γ-secretase inhibitor IX (1 µM) (Calbiochem) was added 15 min before BMP9 treatment. The collected cells were lysed in buffer RLT (Qiagen) for RNA purification.

### Quantitative reverse transcription polymerase chain reaction (qRT-PCR)

The total RNA of HUVECs, fibroblasts and MSCs was extracted using an RNeasy mini kit (Qiagen) following the manufacturer's instructions. cDNA was synthesized with random hexamer primers using a SuperScript III First-Strand Synthesis System for RT-PCR (Life Technologies). Real-time PCR was performed using a TaqMan Gene Expression Master Mix or Power SYBR Green PCR Master Mix, and run on the ABI Prism 7900 sequence detection system with pre-designed primer and probe sets (18S rRNA, 4319413E; Endoglin, Hs00923996_m1; ACVRL1, Hs00953798_m1; BMPR2, Hs00176148_m1; PDGFRB, Hs01019589_m1; Applied Biosystems) or primer sets as follows: HEY1 F, 5′-AGGAGAGTGCGGACGAGAATG-3′; R, 5′-TCGTCGGCGCTTCTCAATTATTCC-3′; TMEM100 F, 5′-CTTTCCCAGAAGTTGGACGA-3′; R, 5′-CCTTGATGGGCTCTTCAGTC-3′; BMPER F, 5′-CCGGCTGAGCCTTGTGTTCTAC-3′; R, 5′-CCCTTCTTGATACTGCACACCCTC-3′; MGP F, 5′-GGCCGCCTTAGCGGTAGTAAC-3′; R, 5′-GGACTTTAGCTCTCCATCTCTGC-3′; NOTCH1 F, 5′-GGAAGTGTGAAGCGGCCAATG-3′; R, 5′-ATAGTCTGCCACGCCTCTGC-3′; NOTCH 2 F, 5′-TGTCGAGATGGCTATGAACCCTG-3′; R, 5′-GCAGCGGTTCTTCTCACAGG-3′; NOTCH 3 F, 5′-TGTCTGCCAGAGTTCAGTGGTG-3′; R, 5′-AGGAGCAGAGGAAGCGTCCATC-3′; NOTCH 4 F, 5′-TTGTCCTCCCTCCTTCTGTTCC-3′; R, 5′-AGAAGTCCCGAAGCTGGCAC-3′; DLL1 F, 5′-TTGCTGTGTCAGGTCTGGAG-3′; R, 5′-TTCTGTTGCGAGGTCATCAG-3′; DLL4 F, 5′-CCTCTCCAACTGCCCTTCAATTTC-3′; R, 5′-ATGAGTGCATCTGGTGGCAAGG-3′; JAG1 F, 5′-TGCCTCTGTGAGACCAACTG-3′; R, 5′-GTTGGGTCCTGAATACCCCT-3′; JAG2 F, 5′-GTGGCAAGAACTGCTCCGTG-3′; R, 5′-TGCCTCTGTGAGACCAACTG-3′; CDH5 F, 5′-GCAGTCCAACGGAACAGAA-3′; R, 5′-CATGAGCCTCTGCATCTTCC-3′. The expression levels of genes were normalized to 18S rRNA as an internal control.

### RBP-Jk luciferase reporter assay

Notch pathway-responsive fibroblasts (RBP-Jk fibroblasts) were established by transducing the lentivirus-based RBP-Jk-responsive luciferase reporter (Cignal Lenti RBP-Jk reporter, Qiagen) into fibroblasts. The RBP-Jk–Luc fibroblasts were seeded into 96-well plates at a density of 20,000 cells per well. The next day, 20,000 cells of HUVECs were seeded into the RBP-Jk–Luc fibroblasts, and stimulated with BMP9, BMP10 (R&D systems), BMP4 (R&D systems), TGF-β (R&D systems), and VEGF (Peprotech). After 16 h incubation, firefly luciferase activity was measured using a Bright-Glo Luciferase Assay System (Promega). The experiments were repeated two times.

### Western blot

HUVECs and fibroblasts were treated with BMP9 (10 ng/ml), BMP4 (100 ng/ml) or TGF-β (10 ng/ml) for 60 min, and lysed with the cell lysis buffer (50 mM Tris-HCl pH 7.5, 150 mM NaCl, 0.1% Triton X-100) containing protease inhibitor cocktail (Roche). Equal amounts of proteins were loaded onto NuPAGE Novex 4-12% Bis-Tris Gels (Life Technologies), and blotted onto a nitrocellulose membrane (Immobilon; Millipore). After blocking with 5% skim milk in Tris-buffered saline containing 0.1% Tween 20 (TBST) for 30 min at room temperature, the membranes were incubated with anti-phospho-Smad1/5/8 antibody (1:1000; CST, 9511L) or anti-β-actin antibody (1:2000; Sigma, AC-74) overnight at 4°C. The membranes were then washed with TBST, incubated with anti-rabbit-IgG-HRP (Amersham) or anti-mouse-IgG-HRP (Amersham) for 1 h, washed again with TBST, and the chemiluminescent signals were detected using ECL Prime (GE Healthcare).
